# Role of Calcium Homeostasis in Modulating EMT in Cancer

**DOI:** 10.3390/biomedicines9091200

**Published:** 2021-09-11

**Authors:** Clark A. Jones, Lori A. Hazlehurst

**Affiliations:** Pharmaceutical and Pharmacological Sciences, School of Pharmacy, West Virginia University, Morgantown, WV 26506, USA; cj0035@mix.wvu.edu

**Keywords:** calcium, EMT, cancer

## Abstract

Calcium is essential for cells to perform numerous physiological processes. In cancer, the augmentation of calcium signaling supports the more proliferative and migratory cells, which is a characteristic of the epithelial-to-mesenchymal transition (EMT). By genetically and epigenetically modifying genes, channels, and entire signaling pathways, cancer cells have adapted to survive with an extreme imbalance of calcium that allows them to grow and metastasize in an abnormal manner. This cellular remodeling also allows for the evasion of immune surveillance and the development of drug resistance, which lead to poor prognosis in patients. Understanding the role calcium flux plays in driving the phenotypes associated with invasion, immune suppression, metastasis, and drug resistance remains critical for determining treatments to optimize clinical outcomes and future drug discovery.

## 1. Introduction

Calcium is one of the most important elemental molecules in the human body; therefore, its regulation is just as crucial. It plays a vital role in many physiological processes, including, but not limited to, muscle contractions, metabolism, phagocytosis, apoptosis, cell division, motility, and signaling [1,2,3,4]. Despite calcium’s expansive role within the body, unbound, cytosolic-free calcium is the only form that can be used for physiological and pathological functions. In order for cells to perform their everyday functions, adequate calcium levels must be met and maintained, leading to the tight regulation of calcium throughout the body. Bones, therefore, act as reservoirs to store excess calcium for utilization when accessible extracellular levels are insufficient in circulation.

Central to calcium homeostasis are the parathyroid hormone (PTH) and calcitonin. When extracellular calcium is low in circulation, PTH is released by the parathyroid glands. This triggers the release of calcium from bone deposits into the bloodstream and the inhibition of calcitonin, a negative regulator of calcium [5]. On the other hand, calcitonin is activated when calcium exceeds its narrow threshold, thereby causing the redeposition of calcium to the bone, along with excretion by the kidneys [5]. Disease states, such as hypercalcemia and osteoporosis, occur when the regulatory factors of calcium homeostasis are not able to function properly [6]. Prolonged uncontrolled fluctuations can lead to severe consequences, including neurological problems, kidney failure, and even death.

**Figure 1:**
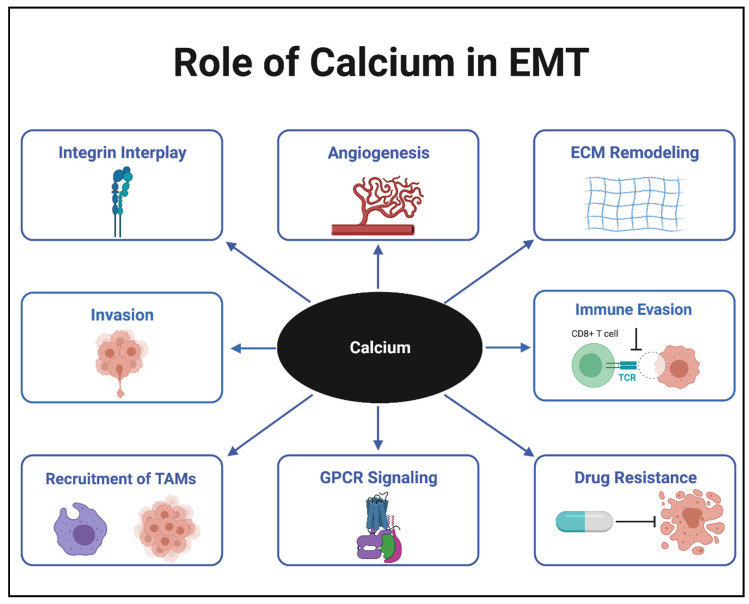
Role of calcium in epithelial-to-mesenchymal transition (EMT). Tumor associated macrophages (TAMs); Extracellular matrix (ECM); G-protein coupled receptor (GPCR); T-cell receptor (TCR).

In cancer cells, mutations and changes in the expression of calcium channels, pumps, and binding proteins have resulted in calcium levels that exceed the typical threshold of normal cells. These elevated calcium levels allow the cells to proliferate and become malignant [7]. Cells that gain the ability to break through the extracellular matrix (ECM) and metastasize to distal portions of the body are said to undergo a process known as the epithelial-to-mesenchymal transition (EMT). The many roles calcium plays in EMT are shown in Figure 1. EMT is a slow, transient process that involves the deterioration of cell–cell junctions and detachment from the basement membrane, where cells lose their polarity. In non-malignant cells, EMT is common for functions such as wound healing, growth, and development. However, in pancreatic, lung, and breast cancer cells, this malignancy process has been shown to lead to poor prognosis and increased tumor progression [8,9,10]. Common epithelial genes that are downregulated for this transition include *E-cadherin*, *claudins*, and *occludins*, which are essential in forming junctions between cells and holding them in place. Mesenchymal genes that are upregulated include *vimentin*, *N-cadherin*, and *matrix metalloproteases* (MMPs), which provide cells with some of the necessary tools to metastasize away from the primary tumor. The reverse process of EMT is called mesenchymal-to-epithelial transition (MET). When nutrients become scarce, cancer cells hijack both the EMT and MET processes to survive. These cells use the EMT process to metastasize from the nutrient-deprived primary tumor and the MET process to recolonize in a distal, nutrient-rich environment. In this environment, they become epithelial, similar to cells attached to a basement membrane, and form cell–cell junctions once more. In this review, the central role calcium plays in EMT are discussed in the following areas: (1) Calcium Channels, (2) GPCR Signaling, (3) Interplay with Integrins, (4) Immune Evasion/Drug Resistance, and (5) Combination Therapy.

## 2. Calcium Channels

In a resting cell, intracellular calcium levels remain close to only 100 nM. This is substantially lower than extracellular calcium concentrations, which range from 1 to 2 mM [7]. Channels and pumps are responsible for maintaining such a tightly regulated concentration within the cell, allowing for a chemical gradient across the cell membrane. The two types of calcium channels that exist in cells are voltage-gated and ligand-gated. Voltage-gated calcium channels (VGCC) cause an influx of calcium into excitable cells, such as neurons [11]. Typically, a quick response is elicited by a VGCC, such as a neuronal signal relay or a muscle contraction. In contrast, ligand-gated calcium channels (LGCCs) induce calcium influx in all other non-excitable cells, and usually generate a much slower and more prolonged effect, which can lead to either cell proliferation or apoptosis, depending on the levels of calcium flux and the cellular context [12]. The variety, localization, and abundance of these channels are tightly regulated, due to their wide range of functionality in different cell types.

The LGCCs are the first step in a cellular process known as store-operated calcium entry (SOCE). This is also the primary means by which malignant cells obtain calcium for cancer progression [13]. Recent studies have shown that proteins such as STIM1, on the endoplasmic reticulum (ER) membrane, and Orai1, on the plasma membrane, play an essential role in the function of these channels. STIM1 is responsible for signaling to the plasma membrane after cellular ER calcium stores are depleted [11]. Orai1 is utilized by the SOCE pathway via pore formation in the plasma membrane, which selectively allows the passage of calcium ions [14]. Another ion channel that allows calcium fluctuations to occur is called a transient receptor potential (TRP). The family of TRP channels is less selective than Orai1, and can allow the passage of other cations, such as sodium, potassium, and magnesium [15]. The literature supports the fact that both subtypes (TRPC and TRPV) are capable of forming complexes with STIM1 and Orai1 to create sustained calcium entry [16,17]. The overexpression of a variety of members from the TRP family has been correlated with increased EMT [18] and poor patient prognosis [19,20].

The dysregulation of these proteins is often linked to multiple cancers, which show increased production and localization to their respective membranes, to accommodate an increased need for calcium. For example, it has been shown that the overexpression of STIM1 in hepatocellular carcinoma leads to increased cytoplasmic calcium and increased cellular proliferation [21]. In addition, there is evidence to support the overexpression of Orai1 in lung cancer correlates with increased cell proliferation, along with poor patient prognosis in the clinic [22]. Calcium channels play the central role in calcium fluctuations in cells, but are not the only means of initiating elevated cytosolic-free calcium. G-protein coupled receptors (GPCRs) are also capable of altering intracellular calcium by means of coordination with SOCE and calcium channels along with crosstalk among additional signal transduction pathways, such as receptor tyrosine kinases (RTK) [23].

## 3. GPCR Signaling

**Figure 2:**
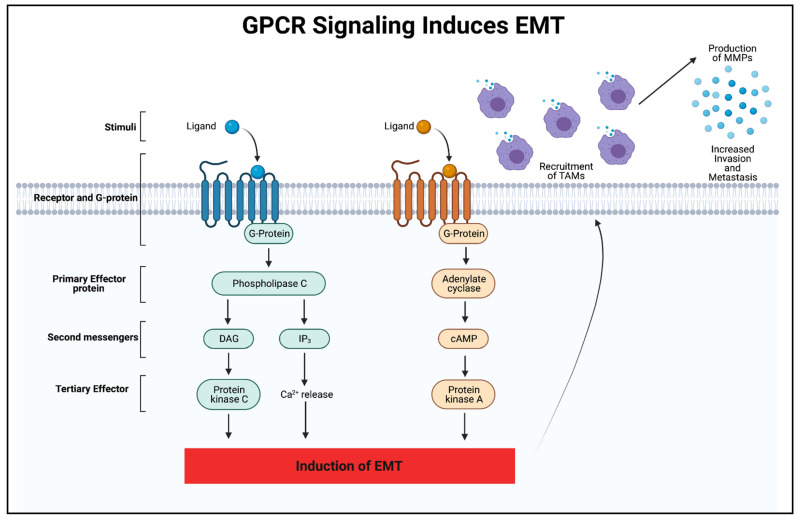
GPCR signaling induces EMT. This schematic illustrates the activation of a GPCR signaling pathway when bound to a ligand such as a chemokine. Both signal transduction pathways have the ability to induce EMT at a transcriptional level by activating genes such as Snail, Twist, and ZEB. GPCR signals that transcriptionally activate EMT genes are capable of recruiting TAMs to the tumor microenvironment. TAMs produce MMPs that allow these cells to invade and metastasize in the EMT process. Diacylglycerol (DAG); Inositol trisphosphate (IP_3_); Cyclic adenosine monophosphate (cAMP).

GPCRs are involved in a plethora of physiological functions, such as the regulation of behavior, the immune system, cell growth, motility, and sensory input [24,25,26,27]. Unfortunately, GPCRs are also involved in numerous disease states, including immune deficiencies, mental and metabolic disorders, lack of sensation, and cancer [28,29,30]. Due to these receptors’ wide-ranging role in so many diseases, it is not surprising that almost half of all FDA-approved drugs target GPCRs for their biological effects. The ligands in cells bind to the GPCR extracellularly to induce a conformation change that activates an intracellular G-protein. This activated G-protein is then capable of signaling through two mechanisms of action, as seen in Figure 2. The first mechanism involves adenylyl cyclase, which produces cAMP as a secondary messenger. The second involves phospholipase C, which is capable of enzymatically hydrolyzing phosphatidylinositol 4,5 bisphosphate (PIP_2_) into diacylglycerol (DAG) and inositol 1,4,5 triphosphate (IP_3_). IP_3_ then binds to the IP_3_ receptor on the ER membrane, causing calcium stores from the ER to empty into the cytoplasm and act as a second messenger to carry out the biological functions [31]. Activation of phospholipase C via G-protein signaling has also been shown to increase intracellular calcium by store depletion and the subsequent activation of TRPC channels [32,33].

Mutations in GPCRs, such as point mutations, overexpression, and silencing, have contributed to either cell death or tumor initiation and EMT. One of the more common alterations discovered after analyzing an mRNA database was GPCR overexpression, coupled with a more frequent mutation rate, which has been found across 20 different cancers, including 45 subtypes, as compared to non-cancerous tissues [34]. These overexpressed GPCRs encompassed all classes, including A, B, C, adhesion, and orphan GPCRs. Since GPCRs can signal to initiate biological processes, such as proliferation and migration, a significant increase in receptors would allow for greater signaling output in these pathways. One example of this phenomenon is found in the family of GPCRs known as chemokine receptors. One role chemokines play in tumorigenesis and EMT is the recruitment of tumor-associated macrophages (TAMs), which leads to the release of MMPs [35]. An illustration of this is shown in Figure 2. These MMPs enhance motility by breaking down proteins in the basement membrane and extracellular matrix, to allow cells to metastasize away from the primary tumor. Recently, several types of the GPCR, protease-activated receptor (PAR), have been found to be upregulated in cancer [36]. Interestingly, the overexpression of PAR was found to induce EMT via TGF-β signaling, leading to the loss of cell polarity [37]. Point mutations in the binding pocket of GPCRs can also be crucial in the viability of cancer cells. The mutations can alter ligand–receptor affinity along with ligand selectivity, which can change the entire signaling pathway through its respective receptor [38]. After analyzing one specific GPCR known as adenosine A_2B_ receptor, a previous study found altered agonist efficacy and potency, with 15 different point mutations [39]. While some of these mutations increased the agonist effect, others reduced or completely eliminated receptor activation, demonstrating how minute changes in the protein sequence can completely transform this GPCR’s capabilities. Recent work has also substantiated GPCRs’ role in the upregulation of EMT transcription factors, such as ZEB, Snail, and Twist, which are involved in numerous transitions, including cell polarity, cytoskeleton remodeling, migration, and invasion [36,40]. It has been challenging to delineate the actual function and various ligands of all GPCRs, due to their extensive crosstalk with the proteins and pathways that perform the vast majority of cellular functions.

## 4. Integrins

**Figure 3:**
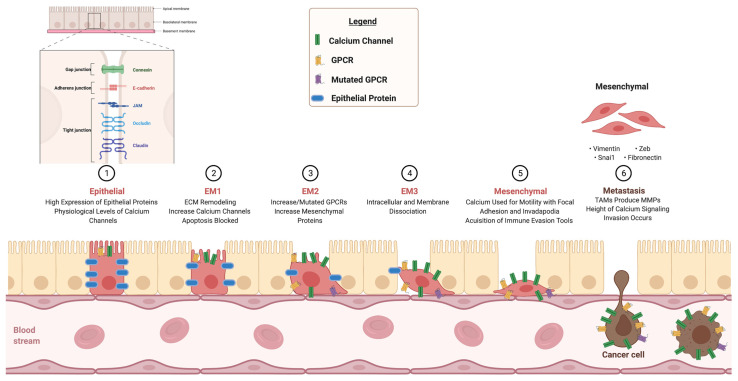
Calcium alterations causative of EMT. This schematic illustrates the role of calcium signaling, which is required for progression from a normal epithelial cell to an EMT phenotype associated with metastatic cancer cells, by showing (1) The starting point of a healthy epithelial cell containing all tight junction proteins and physiological levels of GPCRs and calcium channels. (2) Gradual reduction in junction proteins and increased calcium channel production. (3) Gradual increase in mesenchymal proteins, such as Vimentin and N-Cadherin, and GPCRs acquiring mutations. (4) ECM dissociation through integrins. (5) Increased motility. (6) Invasion of a cancer cell into bloodstream, to metastasize away from primary tumor. Junctional adhesion molecule (JAM); Zinc finger E-box-binding-homeobox (ZEB).

Integrins are transmembrane adhesion receptors that have a primary role in cell–cell and cell–ECM binding, with a secondary role in signaling as well. They are essential for physiological development and are crucial in each step of the development and progression of cancer [41]. The first step in EMT occurs when cell–cell connections are lost and the ECM gains motility. In order for these cells to avoid apoptosis via a process known as anoikis, they rewire their genetic makeup in order to survive on their own, despite losing these connections [42]. There is evidence to suggest that the crosstalk between GPCRs, calcium channels, and integrins, ultimately leading towards EMT, makes this possible (Figure 3). Research studies have shown that certain calcium-sensing G-proteins form signaling complexes with integrins to aid in cell differentiation and movement in cancers. For example, a GPCR with augmented expression from increased extracellular calcium, known as CXCR4, activates a GTPase, known as Rap1, which modulates integrin inside-out signaling by binding talin to β-integrin. This enhances cell–ECM connections, assisting in cellular migration and cytoskeleton rearrangement [43]. One study found that the overexpression of Orai1 was linked to survival of breast cancer cells, a finding that was associated with increased collagen–integrin interactions [44].

In addition, recent studies have found that calcium directly modulates integrin activity. Integrin subunits are formed in the ER and then released into the cytoplasm, and the proper folding of these proteins requires the presence of divalent calcium ions [45]. Additionally, the alpha and beta subunits of integrins cannot localize or activate the conformation in the plasma membrane without the calcium ions. Calcium bound to integrins during transport ensures inactivation until the integrins are membrane bound and the calcium is displaced by additional cations, such as magnesium and manganese, to form an active conformation. Calcium allows the two subunits to dimerize in the ER and translocate to the plasma membrane via endocytosed vesicles for proper functionality [45]. It was also found that manganese and magnesium work in conjunction with calcium to help bridge the integrin–ECM connection, allowing for integrin signaling and cell adhesion [46]. One study found that calcium located at the filipodia of cells was able to modulate the signal transduction process in integrins that were used in migration and invasion for the purpose of tumorigenesis [47]. Whether used in complexes and signaling or directly in cells, calcium has been shown to interact with integrins that regulate normal biological and pathological progressions.

## 5. Calcium in Immune Evasion and Drug Resistance

The most proliferative and aggressive cancer cells possess essential characteristics that enable them to evade immune surveillance [48,49,50,51,52] and become resistant to drug treatment [53,54,55,56,57,58,59,60,61,62]. These characteristics also enable proliferative and aggressive cancer cells to undergo EMT. Cancer cells often contain mutated antigens on the cell surface that are typically marked as non-self by locally activated T cells. Tumor cells with similar antigen presentation are, therefore, disposed of via T-cell infiltration and elimination. The binding of the antigen receptor on the tumor cell to the antigen receptor on the lymphocyte activates phospholipase C, capable of generating IP_3_ and inducing SOCE. This store-operated calcium is used as a second messenger in the lymphocyte to transcriptionally activate NFAT. The lymphocyte is then able to release the calcium-dependent perforin, which disrupts the cancer cells’ membranes, leading to cell death [63]. This process can be initiated in immune cells and blocked in cancer cells by the use of calcium signaling. Cancer cells have manipulated calcium signaling, so that the immune system is no longer able to recognize and dispose of these abnormal cells. The tumor microenvironment plays an essential role in this process, in conjunction with the cancer cells themselves. As previously mentioned in Figure 2, cancer cells are capable of recruiting TAMs through calcium-induced transcriptional regulations to aid in cancer progression. For example, one study found that TAMs colocalized from a wide array of breast carcinomas were involved in the enhancement of tumor progression by producing a chemokine known as CCL18. TAMs located in the breast tumor microenvironment that release CCL18 were able to activate a GPCR known as NIR1. This triggered integrin clustering and ECM remodeling in tumor cells, which were associated with increased calcium signaling through IP_3_ generation, metastasis in multiple sites outside the primary tumor in patients, and poor survival [64]. In addition, by analyzing calcium-dependent and perforin-dependent cytotoxicity in multiple tumor types [65], experiments have shown that the overexpression of Orai1 and excess calcium signaling in cancer cause a reduction in the cytotoxic effect of natural killer cells and the ability of cytotoxic T-lymphocytes to eliminate the cancer. In contrast, the inhibition of Orai1 via siRNA and reduced extracellular calcium decrease cancer cell proliferation and enhance the cytotoxicity of cytotoxic T-lymphocytes and natural killer cells to tumors [65]. While intrinsic factors of tumor cells interacting with the tumor microenvironment can help cells avoid immune detection, extrinsic factors outside these cells can affect cellular processes that lead to drug resistance as well.

Sources of extracellular stress, such as hypoxia, lack of nutrients, and drug pressure, contribute to the induction of EMT by forcing cells to a crossroad of either adapting or dying. Calcium signaling is an important contributor to this process. Small calcium deposits in the tumor microenvironment, known as microcalcifications, have recently been studied as potential links to EMT, and may be a source or product of this increased calcium. One study of prostate cancer found a correlation between microcalcifications and increased bone metastasis [66]. Another study found a similar correlation of microcalcifications and EMT markers, such as CD44 and vimentin, in breast cancer, which could predict a poor prognosis in patients [67]. Intracellular calcium fluctuations have been shown to play a role in epigenetic programs and transcriptional regulation to allow cells to survive in the most extreme cases. For example, sustained calcium entry by means of SOCE has been shown to activate the NFAT/CREB pathway, which induces transcriptional modifications in cells for the progression of tumorigenesis [68]. In contrast, the inhibition of calcium flux causes cancer cells to have increased sensitivity to drug treatment [69]. Further research is still needed to assess whether inhibiting calcium flux results in better patient outcomes. On the cell surface, channels and pumps undergo mutations to improve survival in the face of drug treatments as well. For example, Stim1 was found to work in conjunction with Orai1 to mediate TGF-β, which induces a key transcriptional factor of EMT, called *snai1*, that corresponds with drug resistance [70]. In contrast, efflux pumps play a vital role in cell survival. One study illustrated the role of sorcin (SOluble Resistance-related Calcium-binding ProteIN) overexpression in cancer, which modulates the co-overexpression of ABC (adenosine triphosphate-binding cassette) transporters serving as a survival mechanism by efficiently effluxing drugs out of the cells, in addition to sorcin’s ability to induce EMT [71]. Currently, sorcin is viewed as an oncogene that could be central to multidrug resistance in clinical settings and a viable target for new drug discovery [71]. Furthermore, persister cells in cancer are a common cause of treatment failure and patient relapse. Persister cells make up a very small proportion of malignant cells and have adapted to survive extremely lethal doses of drug treatment [72]. They can also remain in a state of dormancy for extended periods of time, which, in conjunction with a decelerated cell cycle, enables them to acquire mutations that allow for cellular progression [73]. Research has substantiated a consistent trait of these persister cells as having increased calcium signaling in parallel with cytoskeleton remodeling [74]. Understanding the vast array of mechanisms of resistance remains crucial for future drug discovery and optimizing combination therapy.

## 6. Combination Therapy

The heterogeneity of tumors within their microenvironment has proven to be one of the biggest obstacles in treatment. Tumors create diversity through cellular differentiation to obtain a more sustainable environment and prevent their eradication [75,76,77,78,79,80,81]. Evasion of immune surveillance, angiogenesis, ECM remodeling, blockage of apoptosis, and multidrug resistance are all ways in which tumors are able to proliferate and explain why they cannot be captured by a single cellular phenotype. Calcium remains at the center of all of these malignant processes (Figure 1). In order to optimize patient treatment, it is often beneficial to use combination therapy to combat the heterogeneity of the tumor microenvironment. Combination therapy is a practice used for almost all cancers and subtypes. Additional details of how more well-studied cancers, including breast, lung, and colon cancer, respond to combination treatment are presented below. 

Breast Cancer is one of the most common types of cancer and kills more women than any other cancer worldwide [82]. It has been characterized by its many mutations, including human epidermal growth factor 2 (HER2), breast cancer genes 1 and 2 (BRCA1/2), and phosphatase and tensin homolog (PTEN) [83]. Within these mutational subtypes, studies have found alterations of calcium channels and pumps that play a part in the proliferation of breast cancer cells, such as changes in expression and localization, and which allow for sustained calcium signaling [84]. Targeting unique cancer characteristics, such as driver mutations and calcium fluctuations, may be necessary for optimal treatment. Further evidence of this, involving a calcium channel known as TRPV6, which is found to be overexpressed in breast cancer, along with many other cancers, has been found in clinical settings. Clinical trials are ongoing with a novel TRPV6 inhibitor, in conjunction with standard-of-care therapy, for multiple subtypes of breast cancer, since some current treatments, such as tamoxifen, have been shown to negatively affect the TRPV6 channel as well [85]. Many of the current standard-of-care chemotherapy agents also target calcium signaling for the treatment of breast cancer. Cisplatin and tamoxifen act by causing an overwhelming influx of calcium and ER store depletion that leads to cell death, while doxorubicin induces sustained calcium flux, which activates the proapoptotic BIM pathway and causes mitochondrial calcium overload [86].

Lung Cancer is heavily burdened by mutational drivers that make targeted therapy a promising avenue for drug development and impactful in terms of patient outcomes. Some of the more common mutations include epidermal growth factor receptor (EGFR), KRAS, and ALK. EGFR inhibitors remain the most well-developed targeted inhibitors, with strategies to target the most common resistance mutation, T790M, for this disease. Epidermal growth factor (EGF), the ligand for EGFR, was found to play a role in the calcium oscillations essential for EMT [87]. The evidence suggests that the blockage of cells from extracellular calcium via a calcium chelator known as EGTA increases the efficacy of EGFR inhibitors such as afatinib [74]. Immunotherapies are also on the rise in cancer treatment. One study found that blocking calcium channels can suppress the transcription of programmed death-ligand 1 (PD-L1) and enhance natural killers cells’ ability to eliminate the cancer [88]. Chemotherapies that target calcium signaling, similar to those used against breast cancer, such as cisplatin and doxorubicin, were found to be synergistic in combination with EGFR inhibitors (gefitinib and erlotinib) and to prolong patient survival in the clinic [89].

Colon Cancer is the third most common cause of cancer-related death. Little is known about the causes, but the most prominent driver mutations include adenomatous polyposis coli (APC), BRAF, and KRAS [90]. SERCA channels are often targeted to deplete ER calcium stores. One SERCA channel blocker used in combination with standard-of-care drugs for colon cancer is called thapsigargin [91]. Stim1 and Orai1 inhibitors have also been used to increase patient survival rates [91]. In addition, one standard-of-care drug for colon cancer, known as 5 Fluorouracil (5-FU), acts by modulating calcium itself. 5-FU was found to signal through a calcium-dependent pathway in order to induce apoptosis [92].

By incorporating the role of calcium signaling in the development of new drugs, therapies can evolve to optimize cancer treatment. This has been shown in the treatment of other cancers, such as prostate, pancreatic, and glioblastoma. As with lung cancer treatment, cisplatin and paclitaxel alter calcium homeostasis and remain an effective first-line treatment for prostate cancer that has an overexpression of TRPV channels [93,94]. Pancreatic cancer is similar to colon cancer, with the infamous KRAS mutation and the overexpression of Orai1. An additional similarity between the two malignancies is the use of the calcium-induced apoptosis treatment of 5-FU [95]. Even glioblastoma, one of the most lethal cancers, has started to be treated with calcium-altering therapies. T-Type calcium channel blockers, combined with temozolomide (TMZ), are in the early stages of clinical trials for use in the treatment of high-grade gliomas [96,97]. A comprehensive table, containing calcium alterations across multiple cancers and calcium pathway-targeting therapies, is shown in Table 1. Combination therapy has become especially important with the increasing knowledge of EMT and multidrug-resistant mechanisms. While some standard-of-care drugs directly target calcium pathways, targeting multiple broad mutations of the heterogenous population within the tumor microenvironment may effectively reduce drug resistance and improve long-term patient outcomes.

## 7. Conclusions

Cancer constantly evolves, with mutational burdens that are continually being explored. Therefore, understanding commonalities that lead to the development of a more malignant EMT phenotype is essential for drug discovery and the expansion of combination therapies. A plethora of evidence has substantiated the fact that calcium plays a critical role in the EMT process, leading to immune evasion and drug resistance. Calcium modulates cellular functions by inducing alterations in calcium channels; interacting with receptors; and remodeling the ECM signaling pathways, transcription, and epigenetics. This allows normal cells to transition into cancer cells. Insights into specific changes at a transcriptional and protein level will aid in the construction of new targeted therapies to optimize the eradication of the heterogenous tumor population. Since the EMT is a transient process, the reversal of a malignant phenotype or the blockage of its progression would also be a viable option for improving patient outcomes. Calcium signaling and its crosstalk with a multitude of additional signaling pathways can obscure correlative data as causative evidence. As a result, further research is needed to improve knowledge on delineating calcium’s many roles in the EMT process and cancer progression, to help those faced with this deadly disease.

## Figures and Tables

**Table 1:** Calcium changes by cancer type as a result of therapies targeting calcium pathways.

Cancer Type	Mutational Drivers	Calcium Alterations	Calcium Altering Therapies	References
Lung	EGFR	↑Orai1	Cisplatin	
	KRAS	↑TRPC1,3,4,6	Doxorubicin	
	ALK		Afitinib	[22,74,87]
Breast	HER2	↑TRPV6	Cisplatin	
	BRCA1/2		Tamoxifen	
	PTEN		TRPV6 Inhibitor	[83,85,86]
Colon	APC	↑Stim1/Orai1	Stim1/Orai1 Inhibitors	
	KRAS	↑SERCA2	Thapsigargin	
	BRAF	↑IP_3_	5-FU	[90,91,92]
Prostate	FOXA1			
	TP53	↑TRPV1	Paclitaxel	
	PTEN	↑TRPV2	Cisplatin	[93,94]
Pancreatic	KRAS	↑TRPM7	5-FU	
	TP53	↑Orai1		[95,98]
Glioblastoma	LOH Loss	↑TRPV1	T-Type Calcium Channel Blocker Combined with TMZ	
		↑TRPV2		[96,97]

↑indicates increased expression.

## Data Availability

Not applicable.
